# Climate-driven changes of global marine mercury cycles in 2100

**DOI:** 10.1073/pnas.2202488120

**Published:** 2023-01-03

**Authors:** Yujuan Wang, Peipei Wu, Yanxu Zhang

**Affiliations:** ^a^School of Atmospheric Sciences, Nanjing University, Nanjing, Jiangsu 210023, China

**Keywords:** mercury, climate change, MITgcm, IGSM, methylmercury

## Abstract

Human exposure to monomethylmercury (CH_3_Hg), a potent neurotoxin, is principally through the consumption of seafood. The formation of CH_3_Hg and its bioaccumulation in marine food webs experience ongoing impacts of global climate warming and ocean biogeochemistry alterations. Employing a series of sensitivity experiments, here we explicitly consider the effects of climate change on marine mercury (Hg) cycling within a global ocean model in the hypothesized twenty-first century under the business-as-usual scenario. Even though the overall prediction is subjected to significant uncertainty, we identify several important climate change impact pathways. Elevated seawater temperature exacerbates elemental Hg (Hg^0^) evasion, while decreased surface wind speed reduces air–sea exchange rates. The reduced export of particulate organic carbon shrinks the pool of potentially bioavailable divalent Hg (Hg^II^) that can be methylated in the subsurface ocean, where shallower remineralization depth associated with lower productivity causes impairment of methylation activity. We also simulate an increase in CH_3_Hg photodemethylation potential caused by increased incident shortwave radiation and less attenuation by decreased sea ice and chlorophyll. The model suggests that these impacts can also be propagated to the CH_3_Hg concentration in the base of the marine food web. Our results offer insight into synergisms/antagonisms in the marine Hg cycling among different climate change stressors.

## Significance

One concern caused by the changes in the ocean due to climate change is the potential increase of neurotoxic methylmercury content in seafood. This work quantifies the impact of global change factors on marine mercury cycles. The air–sea exchange is influenced by wind speed weakening and solubility drop of mercury due to seawater warming. The decreased biological pump shrinks the methylation substrate and causes weaker methylation. The advantageous light environment resulting from less attenuation by sea ice and phytoplankton increases the photodegradation potential for seawater methylmercury. Responses of seawater methylmercury can propagate to biota, which is also modulated by the changes in anthropogenic emissions and ocean ecology. Our results offer insight into interactions among different climate change stressors.

Methylmercury (CH_3_Hg) is the most toxic form of mercury (Hg) compounds that cycles around the globe and bioaccumulates in the marine food webs (1). Human exposure to this potent neurotoxin most commonly occurs through seafood ingestion and raises serious concerns in public health (2). The Minamata Convention, an international legally bound treaty that took effect in 2017, aims to reduce anthropogenic Hg emissions and related health risks (https://www.mercuryconvention.org/). However, its effectiveness is compounded by the changing climate and marine environment (3–5).

The CH_3_Hg found in marine ecosystems mostly came from the atmospheric deposition of inorganic Hg (6, 7). Under particular biogeochemical conditions, inorganic Hg in seawater can be methylated by microorganisms in situ fueled by the remineralization of sinking organic particulate carbon (8, 9). Demethylation of the formed CH_3_Hg also takes place photochemically and biologically (10–12). The resulting bioavailable CH_3_Hg diffuses into the ecosystem and can bioaccumulate along the marine food web efficiently (1, 4, 13).

The ocean is predicted to undergo a profound change in this century due to climate change (14, 15). Human-induced excessive radiative forcing directly warms the ocean, reduces the sea ice content, and the increasing atmospheric CO_2_ concentrations acidify the ocean (14, 16). Variations in upper-ocean temperature will intensify stratification and thus reduce vertical mixing (17, 18). These changes further modulate ocean biogeochemistry indirectly with perturbations on light availability, biological productivity, carbon cycling, and ecosystem functioning (19–22).

Predicting the responses of marine Hg cycles to climate change remains challenging, requiring an understanding of multiple influencing mechanisms and a comprehensive process-based model (3). Zhang et al. (5) have hypothesized that the changing ocean biogeochemistry, particularly ocean acidification, can exacerbate CH_3_Hg production and enhance CH_3_Hg entry into the marine ecosystem. However, this work did not deal with the influence of physical factors, especially the surface wind, sea ice content, light environment, and temperature. Here, we quantify the twenty-first-century climate-related effects on marine Hg cycling under a worst-case scenario devoid of additional efforts to restrain human perturbations (i.e., RCP8.5, a.k.a., “business as usual”) by employing a global ocean model for Hg (9) coupled with the Darwin ocean ecosystem model (23) that is driven by the results of an intermediate earth system model (*SI Appendix*, Fig. S5 and *Methods*). We consider a simulation running from 1991 to 2000 as the present-day or base scenario. To disentangle the impact of different influencing factors, we conduct a set of sensitivity experiments by alternatively changing one factor to the condition in 2091 to 2100 with other factors the same as the present-day scenario. These factors include ocean physics such as ocean current velocity and mixing, seawater temperature, shortwave radiation, near-surface wind speed, and sea ice coverages, ocean biogeochemistry such as seawater chlorophyll (Chl), dissolved organic carbon (DOC), particulate organic carbon (POC), and organic carbon remineralization rates (OCRR); and ocean ecology such as plankton community structure. We also differentiate the ocean biogeochemistry changes caused by ocean physics and acidification (i.e., increase in seawater *p*CO_2_) (*SI Appendix*, Table S2). The input of Hg from the atmosphere is held constant for all the present-day and future scenarios, so the difference between them could represent the impacts of individual changing ocean environmental factors.

## Results and Discussion

### Inorganic Hg.

We calculate a modeled global average total inorganic Hg concentration in the surface layer (euphotic zone, upper 100 m) of 0.51 ± 0.16 picomolar (pM = 10^−12^ mol/L) at present-day, consistent with the observed values in both magnitudes and spatial distributions (*SI Appendix*, Fig. S1*B*). There are three tracers of inorganic Hg in the model, which are dissolved phase elemental Hg (Hg^0^) and divalent Hg (Hg^II^), and particulate phase Hg (Hg^P^) (9). The bidirectional exchange of Hg^0^ at the air–sea interface is simulated over uncovered sea surface area based on the concentration gradient across the interface and piston velocity calculated as a function of wind speed. In the oceanic mixed layer, both photochemical and biological redox conversions between Hg^0^ and Hg^II^ are modeled. The dark oxidation of Hg^0^ is also included (refer to *SI Appendix*, Table S5 for detailed description).

**Fig. 1.**
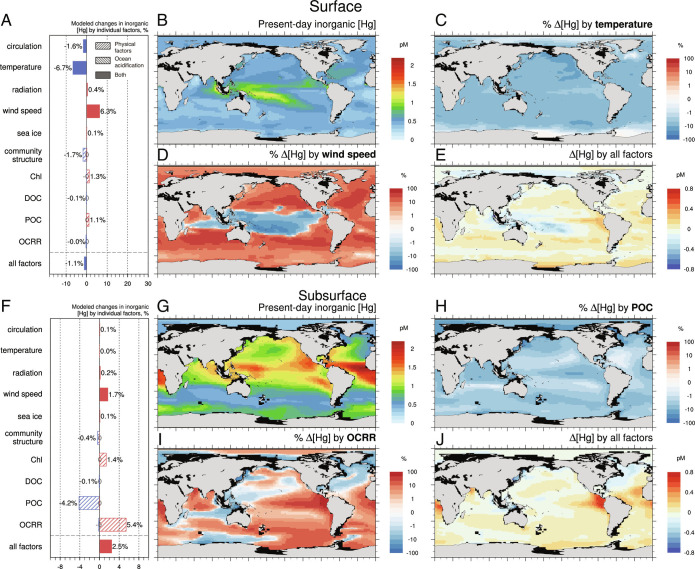
Modeled effects of climate change on inorganic Hg concentration in the surface (*A–E*) and subsurface (*F*–*J*) ocean. The colors of the bars, blue and red, indicate a positive and negative change, respectively. “/” and “\” stripes in the bars represent the effects of biogeochemical factors driven by physical factors (e.g., seawater temperature, ocean circulation, and radiation) and *p*CO_2_-related ocean acidification, respectively. The changes associated with ocean acidification are noted in gray numbers (values less than 0.5% are marked as “0”). Model results of present-day inorganic Hg concentration (*B* for surface and *G* for subsurface) and differences resulted from all factors between 2100 and present-day (*E* surface and *J* subsurface). The percent changes of inorganic Hg between 2100 and present-day contributed by individual factors in the surface ocean: (*C*) temperature and (*D*) wind speed, and in the subsurface ocean: (*H*) POC and (*I*) OCRR. Chl denotes seawater chlorophyll concentrations.

We find from the results of sensitivity experiments that seawater temperature and near-surface wind speed have the largest influence on the total inorganic Hg concentration in the surface ocean, compared to other factors (Fig. 1*A*). On the one hand, the overwhelming rise of seawater temperature over the globe (*SI Appendix*, Fig. S2*A*) reduces the solubility of gaseous elemental Hg [(Hg^0^(g)] (within the 10 to 35 °C range, a 1 °C rise would lead to a 2.5 to 2.9% drop) (24), facilitating Hg^0^ to escape from the ocean. The net evasion flux of Hg^0^ is increased by 1.9% in this way, causing a global decrease of 6.7% in the modeled surface total inorganic Hg concentrations (Fig. 1*C*). On the other hand, the near-surface wind speeds are predicted to be weakened over regions other than the tropical Pacific, Bay of Bengal, and the equatorial Indian Ocean (*SI Appendix*, Fig. S2*B*), resulting in a rise in the surface total inorganic Hg concentration. Indeed, intensified wind in certain areas could cause a faster air–sea exchange velocity and subsequent Hg^0^ evasion. Yet on a global basis, the net evasion flux of Hg^0^ is predicted to decrease by 2.9% due to wind speed changes, and this leads to a global increase of 6.3% in the modeled surface total inorganic Hg concentrations (Fig. 1*D*). To sum up, perturbations to air–sea exchange from climate change (warming ocean temperatures and lower wind speeds) can have contrasting effects on total inorganic Hg concentrations in the surface ocean. The model suggests these effects are about equal in magnitude and opposite in sign. Other factors have a much smaller effect on the total inorganic Hg concentration in the surface waters.

A key process in transporting inorganic Hg to subsurface waters is particle sinking (i.e., biological pump), which largely shapes the vertical profile of seawater Hg concentrations. The Hg^P^ concentration is calculated from dissolved Hg^II^ concentration using a partition coefficient and local POC level. Then with the export of organic particles in the water column, Hg^P^ sinking flux is calculated. In the subsurface layer (100 to 1000 m, below the mixed layer including the major thermocline), the simulated global average total inorganic Hg concentration is 0.98 ± 0.38 pM, consistent with observations. The spatial distribution of total inorganic Hg in the subsurface ocean from our model is close to available observations as well, indicating that our model also captures the vertical distribution of observations (*SI Appendix*, Fig. S1*D*).

We find that POC concentration and OCRR are modeled to have a more pronounced effect in the subsurface ocean, and temperature and surface wind speed are not important influencing factors (Fig. 1*F*). In response to future ocean warming, remineralization shoals and the export of POC to deeper ocean decrease mainly in regions that are nutrient-limited (e.g., the Peruvian upwelling system) (25). These changes predicted by the Darwin model can be attributed to a higher temperature that intensifies ocean stratification and promotes remineralization at shallower depths, which, together with reduced primary production, leads to a reduction in the export of POC. The remineralization in the deeper waters is then decreased (26). The reduction of sinking matter may leave entrained Hg higher up in the surface water (27) and subsequent more evasion out of the ocean. In the case of reduced POC sinking, a 21.3% decrease in Hg^P^ sinking flux and a 2.0% increase in Hg^0^ evasion flux are modeled, resulting in a decrease in the subsurface inorganic Hg of 4.2% (Fig. 1*H*). In contrast, the weakened remineralization of organic carbon at depth prevents the methylation of inorganic Hg^II^, leading to a 5.4% buildup of subsurface inorganic Hg (Fig. 1*I*). These results demonstrate that future changes in the biological pump could affect the cycling of Hg in different ways. Overall, we find the seawater levels of inorganic Hg, the substrate of CH_3_Hg, are relatively less impacted by climate changes.

### Surface Seawater CH_3_Hg.

The seawater CH_3_Hg concentration in the surface ocean reflects a dynamic balance between methylation and demethylation processes and alteration in either of them affects CH_3_Hg (28). The model captures the observed spatial distribution of total methylated Hg [MeHg, i.e., CH_3_Hg and (CH_3_)_2_Hg] in the surface ocean, especially the latitudinal pattern (*SI Appendix*, Fig. S1*A*). The formation rates of methylated Hg [CH_3_Hg and (CH_3_)_2_Hg] are parameterized proportionally to microbial respiration/remineralization of organic carbon in the model, which is influenced by ocean warming (8, 9, 25). Demethylation includes both photochemical and dark processes. The rate constants of photochemical reactions are scaled by the shortwave radiation intensity attenuated by ocean pigments (e.g., chlorophyll and DOC). The dark demethylation rate is calculated as a function of seawater temperature (9) (refer to *SI Appendix*, Table S5 for detailed description).

**Fig. 2.**
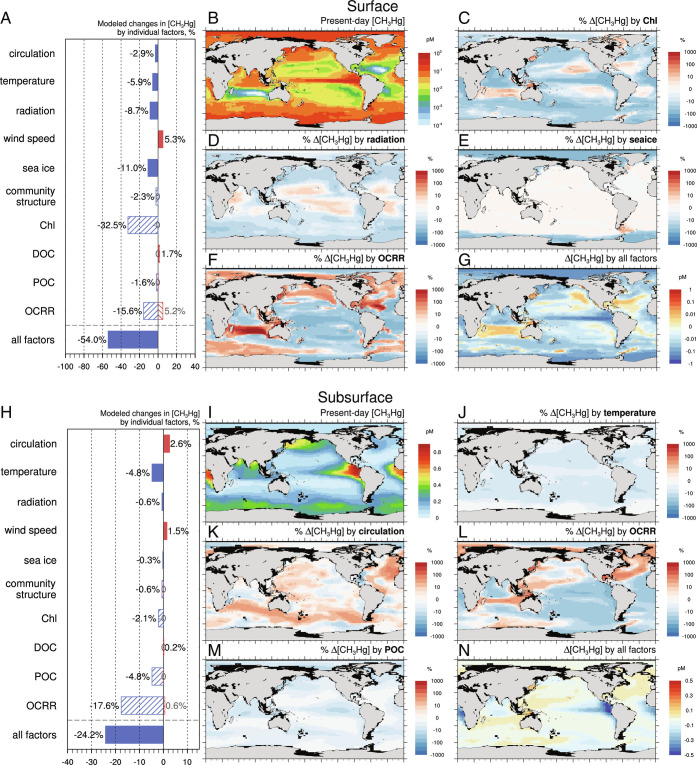
Modeled effects of climate change on CH_3_Hg concentration in the surface (*A*–*G*) and subsurface ocean (*H*–*N*). Model results of present-day seawater CH_3_Hg concentration (*B* surface and *I* subsurface) and differences resulted from all factors between 2100 and present-day (*G* surface and *N* subsurface). Panels *C*–*F* and *J*–*M* are for individual factors similar to Fig. 1.

Among the results of our sensitivity experiments, the most significant factor for future surface water CH_3_Hg changes is chlorophyll concentrations (Fig. 2*A*). A considerable decrease (33%) in global average surface ocean CH_3_Hg concentrations is simulated under the future scenario where there occurs a broad decrease in projected chlorophyll concentrations relative to present days (*SI Appendix*, Fig. S2*F*). Earlier studies of in situ measurements have reported a global-scale decline in surface chlorophyll over the past century (29). This allows more light penetration and triggers a negative feedback loop as increased light also enhances productivity and potentially chlorophyll, which is considered by the Darwin ecosystem model we use here (23). The global mean chlorophyll concentrations are still predicted to reduce by 54% from 2.52 mg m^−3^ to 1.17 mg m^−^^3^ in 2100, close to other estimations by Olonscheck et al. (30) and Hofmann et al. (31). We attribute the modeled decreases in the future seawater CH_3_Hg associated with chlorophyll to enhanced photo-induced degradation of CH_3_Hg. As the incident radiation undergoes the attenuation of chlorophyll, less chlorophyll in the future surface ocean thus allows more light penetration, thereby enhancing the decomposition of CH_3_Hg. A set of sensitivity simulations forcing the model with different future chlorophyll changes results in nonlinear and asymmetrical effects: doubling or halving the current-day chlorophyll profile would lead to a 35% increase or 24% decrease in the CH_3_Hg concentration, respectively. This suggests that the chlorophyll-related effect on seawater CH_3_Hg depends on the global distribution of chlorophyll under climate change scenarios (Fig. 2*C*).

Rises in the future DOC concentration (*SI Appendix*, Fig. S3), however, have a modest effect on photodemethylation processes due to a lower specific attenuation coefficient of light than chlorophyll in our model. There is also a positive feedback loop as the increased light decomposes DOC and further increases the incident light, which is, however, not included in the ecosystem model (23). As a result of future anthropogenic interference on atmospheric transparency (32), the future changes in shortwave radiation incident at the earth’s surface also decompose surface ocean CH_3_Hg and would result in an 8.7% decrease. However, it has a different spatial pattern with the effect of chlorophyll (*SI Appendix*, Fig. S2*B*): at high latitudes, surface solar radiation increases, enhancing the photodecomposition of CH_3_Hg, while surface solar radiation decreases in the tropical regions (Fig. 2*D*). Due to higher CH_3_Hg concentration in the polar surface ocean resulting from lower annual mean shortwave radiation (9), the “brightening” in this region would reduce surface CH_3_Hg more significantly than in the lower latitudes.

The dark demethylation via biotic/abiotic processes is presumed to be temperature-dependent in the model (9, 33) (refer to *SI Appendix*, Table S5), and the projected ocean warming results in a 5.9% decrease in CH_3_Hg (Fig. 2*A*). Another major physical factor influencing surface ocean CH_3_Hg concentration is the massive loss of sea ice in the polar oceans, which results in an 11% decrease of CH_3_Hg in the future. Since ice cover shields seawater CH_3_Hg against photodegradation, sea ice extent reduction owing to climate change can cause more CH_3_Hg to break down (34) (Fig. 2*E*). Besides, the loss of sea ice cover for extensive areas of the ocean favors the air–sea exchange of volatile dimethylmercury [(CH_3_)_2_Hg] (35), which would result in a 21% decrease in the future surface seawater (CH_3_)_2_Hg and also promote the loss of seawater CH_3_Hg. Recent measurements show lower Arctic seawater (CH_3_)_2_Hg concentrations, suggesting likely less importance for seawater (CH_3_)_2_Hg evasion (36). Schaefer et al. (37) also suggested that the direct evasion flux of (CH_3_)_2_Hg from sea ice is close to that from seawater, which also confirms the influence of Arctic sea ice regime shift on the long-term trends of methylmercury. The influence of other factors on future seawater (CH_3_)_2_Hg concentration is in line with those of seawater CH_3_Hg.

The results of sensitivity experiments suggest that future changes in OCRR will also have some impact on CH_3_Hg concentrations in the surface ocean (Fig. 2*F*). Future OCRR is predicted to decline in major upwelling zones due to increased stratification and subsequent lower primary production, such as the tropical Pacific and the eastern boundary of the South Atlantic subtropical gyre, where waters are supposed to be nutrient-rich with high biological productivity (*SI Appendix*, Fig. S3). This denotes decreased microbial methylation activity and will lead to a decrease in the surface CH_3_Hg concentration. This possible variation is consistent with what previous studies have pointed out (5). Although there are several regions with a simulated increase of OCRR and favorable methylation potential, i.e., the Indian Ocean gyre and the North Atlantic gyre, a reduced OCRR and methylation are modeled in upwelling regions as mentioned above. Globally, the model predicts that future OCRR changes reduce CH_3_Hg concentration in the surface ocean by 10% in total. The added effects of ocean acidification are also quantified (*Methods*). As elevated seawater *p*CO_2_ promotes diffusive uptake of CO_2_ by phytoplankton, this saves the energy consumption of CO_2_ uptake and diverts the energy to other activities such as photosynthesis, which potentially increase the OCRR (38–40). This CO_2_ fertilization effect counteracts the physical factor-caused changes (*SI Appendix*, Fig. S3, third row).

### Subsurface Seawater CH_3_Hg.

In the subsurface ocean, the distribution of seawater CH_3_Hg is influenced by a variety of factors such as the vertical and horizontal transport of CH_3_Hg and in situ methylation/demethylation activities. The degradation of CH_3_Hg below euphotic layers involves both biotic and abiotic pathways (41, 42). This degradation rate is assumed to be influenced by ambient temperature in the model (9). Future seawater temperature rise may promote demethylation and simulate a 4.8% CH_3_Hg decrease (Fig. 2*J*), similar to the surface ocean. Future changes in ocean circulation may affect the vertical transport of Hg (43), and an overall weakened upwelling and notable mixing layer shoaling in the subtropical ocean (18) is simulated to lead to a 2.6% rise in subsurface CH_3_Hg and a 2.9% drop in surface CH_3_Hg (*SI Appendix*, Fig. S2 *E* and *H*).

The model estimates that the future spatially inhomogeneous variations in OCRR will affect seawater CH_3_Hg below the top 100 m, resulting in a substantial decrease of 18% in the global mean. This can be attributed to the significant decline in CH_3_Hg production in regions that exhibit the highest subsurface CH_3_Hg concentration, i.e., eastern tropical Pacific and Benguela upwelling system as noted by Zhang et al. (9) (Fig. 2*L*). In these major productive zones, future drops in OCRR (*SI Appendix*, Fig. S3) indicate the reduction in microbial methylation, which causes the decrease of subsurface CH_3_Hg. OCRR changes induced by ocean acidification have a relatively smaller impact on subsurface CH_3_Hg (~1%), consistent with previous studies (5). The associated lowered transport of POC (*SI Appendix*, Fig. S3) is simulated to reduce the seawater CH_3_Hg concentration in the subsurface ocean by 4.7%. As was pointed out in the previous discussion of subsurface inorganic Hg, the substrate Hg^II^ required for methylation would be reduced, thereby inhibiting the formation of CH_3_Hg (Fig. 2*M*).

### Plankton CH_3_Hg.

**Fig. 3.**
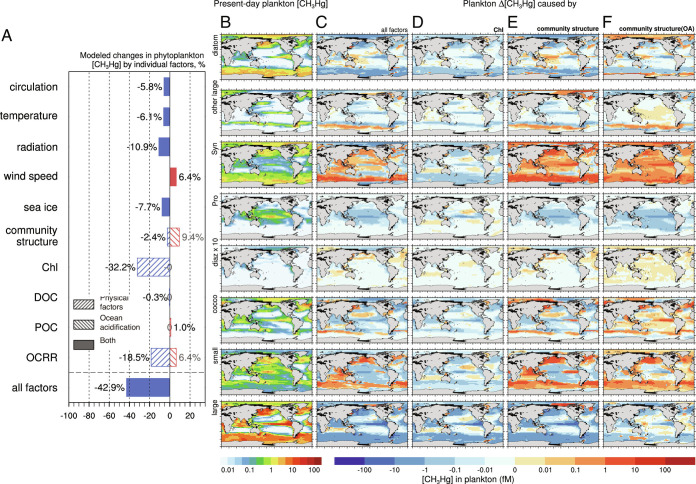
Modeled effects of climate change on surface CH_3_Hg concentration in phytoplankton (*A*). For each plankton group (diatom, other large, *Synechococcus, Prochlorococcus*, diazotrophs, coccolithophores, small zooplankton, and large zooplankton), column *B* is for the model results of present-day plankton CH_3_Hg concentration, *C* is the differences between 2100 and present-day caused by all factors, *D* Chl effects, *E* planktonic community structure effects (including the influence of physical changes and ocean acidification), *F* planktonic community structure effects caused by ocean acidification only.

Biological uptake of CH_3_Hg by pelagic food webs is presumed to be an instantaneous process in the model (9). CH_3_Hg concentration in phytoplankton is calculated based on the volume concentration factor (VCF) with seawater CH_3_Hg concentration. VCF varies as a function of phytoplankton cell diameter (*d*) and seawater DOC concentration (13, 44) (refer to *SI Appendix*, Table S5). The phytoplankton CH_3_Hg concentration is thus directly limited by the availability of CH_3_Hg in seawater. Hence, simulated plankton CH_3_Hg concentrations adjust similarly to seawater CH_3_Hg concentrations in response to climate change-driven alterations (Figs. 2*A* and 3*A*). The effect of reduced chlorophyll on facilitating photodemethylation, which is essentially connected with seawater CH_3_Hg changes as discussed above, reduces the surface phytoplankton CH_3_Hg concentration by 32% (Fig. 3, column *D*). The chemical composition and origin of DOC are known to impact the bioavailability of CH_3_Hg (45) but are not considered in this study limited by the availability of DOC composition data.

Anticipated changes in plankton community dynamics are also a key determining factor of future CH_3_Hg bioaccumulation. After the uptake of phytoplankton, the CH_3_Hg trophic transfer to zooplankton is modeled in association with processes including zooplankton grazing, excretion, and mortality (9) (refer to *SI Appendix*, Table S5). Our simulation reveals that this will increase the global average concentration of CH_3_Hg in all phytoplankton by 7.0%, including a 9.0% increase attributable to ocean acidification and a 2.0% decrease to physical factors, consistent with earlier findings in Zhang et al. (5) (Fig. 3*A*). Large eukaryotic groups such as diatom (*d* = 12 μm as represented by the Darwin model, the same hereinafter) and other large (10 μm) are modeled to migrate to the polar regions due to ocean warming, which could cause up to a factor of two changes in polar regions (Fig. 3 *E*–*F*). The projected increase of *Synechococcus* (*d* = 1.8 μm) under the impact of ocean acidification also increases its CH_3_Hg quota in most regions (Fig. 3, columns *E, F,* and *SI Appendix*, Fig. S4) (46). According to the model assumption on CH_3_Hg uptake by phytoplankton, as the cellular surface-area-to-volume ratio (inversely proportional to the diameter) increases, this favorable transition to more *Synechococcus* in the community structure would promote phytoplankton to uptake more CH_3_Hg, especially in high latitudes (13). On the contrary, the transfer from *Prochlorococcus* (*d* = 0.6 μm) to *Synechococcus* causes a decrease in plankton uptake of CH_3_Hg in the tropical regions as the former is even smaller in cell size. Subsequently, the CH_3_Hg concentration in the small zooplankton (*d* = 30 μm), which prefers to feed on smaller phytoplankton (i.e., *Prochlorococcus* and *Synechococcus*), is projected to increase substantially by 65.7% under the influence of planktonic community changes. However, for the large zooplankton (*d* = 300 μm), which has higher CH_3_Hg concentrations due to slower elimination, the loss of diatoms in the equatorial Pacific and the Southern Ocean disrupts the CH_3_Hg transfer to the large zooplankton. The changes within the food web dynamics bring about a great 71.0% decline in the CH_3_Hg concentration of large zooplankton. Compared with the changes in the availability of seawater CH_3_Hg, plankton community changes may have an overall smaller global impact but could affect the biogeographic distribution and play a much more important role at basin scales.

### Coastal Area Changes.

**Fig. 4.**
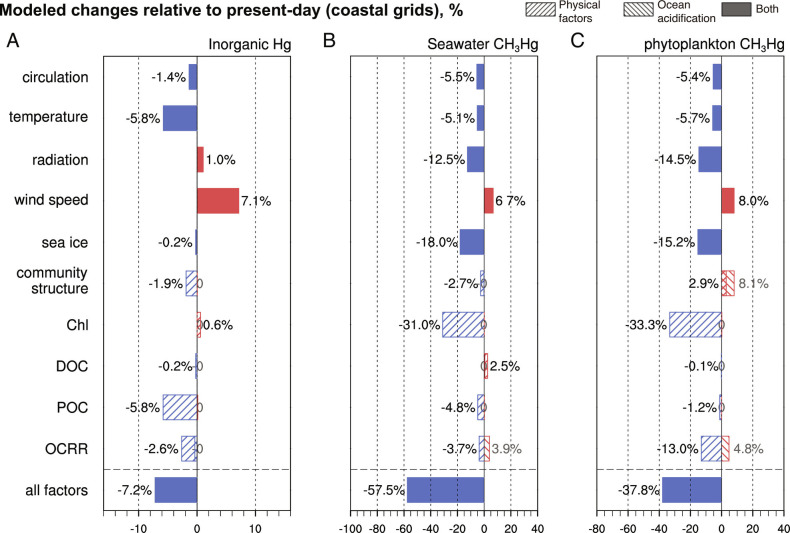
Modeled effects of climate change on average Hg concentrations in coastal grids in the surface ocean. (*A*) Inorganic Hg concentration, (*B*) seawater CH_3_Hg concentration, and (*C*) CH_3_Hg concentration in phytoplankton.

Since CH_3_Hg exposure is tightly associated with dietary seafood, the response of coastal area Hg contamination to future climate change is of great intrigue. Indeed, coastal fishing and aquaculture are becoming a more dominant source of human seafood consumption worldwide, especially the Asian countries (Food and Agriculture Organization, https://www.fao.org/documents/card/en/c/ca9229en/). We use results from different scenario simulations to obtain changes in mean Hg concentrations at coastal grid points and find that the responses are mostly consistent with those of the global mean (Fig. 4). However, there are differences in the effects of POC on surface inorganic Hg concentrations at the coastal scale. The future enrichment of surface seawater POC mainly located in coastal areas signals the weakening of POC export in this region, depriving the efficient downward transport of particulate Hg (*SI Appendix*, Fig. S3*B*). Under such circumstances, the retention of inorganic Hg in the surface layer allows the facilitation of sea–air exchange processes and the loss of inorganic Hg from seawater. Besides, compared to a 7% increase in the global mean, an 11% increase in surface phytoplankton CH_3_Hg at the coastal scale indicates that future changes in plankton community structure are more favorable for CH_3_Hg bio-uptake near shore.

The effects of excessive nutrient discharge to estuaries and coastal waters, for example, include direct DOC enrichment, which may either increase the activity of CH_3_Hg subjected to photodegradation by forming complexes with CH_3_Hg or inhibit photodemethylation by attenuating solar radiation (47). Besides, the nearshore plankton blooms resulting from eutrophication are found to dilute biota CH_3_Hg concentration (48). We perform a complementary simulation that includes the effects of future changes in riverine nutrient inputs based on the NEWS 2 dataset in the marine ecosystem model (49, 50). The export of nutrients (C, N, P, Si) under the future scenario and the present-day condition is separately fed into the Darwin ocean ecosystem model to derive the changes in ocean biogeochemistry (*Methods*). Ocean physics and other parameters are kept the same to highlight the effects of eutrophication.

The modeled Hg species concentration at coastal areas from future nutrient loading does not differ significantly from those of present days at a global scale but with significant regional variability (*SI Appendix*, Table S4 and Fig. S7). For example, the nearshore regions of East Asia, the Bay of Bengal, and the Arctic are subject to a more prominent increase in surface seawater CH_3_Hg concentrations due to the faster increase in riverine nutrient load, while decreasing trends are modeled for the Arabian Sea and the North Atlantic (*SI Appendix*, Fig. S7). Marine productivity changes in these regions alter microbial Hg methylation (*SI Appendix*, Fig. S6). The changes in pigments, such as chlorophyll and DOC, also influence the photodegradation of CH_3_Hg. Furthermore, the Hg-related risks for coastal eutrophication are possible to be of greater concern when combined with future physical changes in the coastal areas and the change of riverine Hg discharges, which could be a considerable source of the Hg burden in coastal oceans (51). These results, however, should be interpreted with caution because the global model used in this study, limited by coarse spatial resolution, does not adequately resolve estuarine and basin-scale processes, some of which may act to alter the feedback expounded here.

### Uncertainties.

Model uncertainty arises from several aspects. The model does not account for the formation and deposition of CH_3_Hg in estuary sediments and inputs of riverine Hg. The resuspension of CH_3_Hg from sediments as an extra source to ocean water, particularly coastal waters, is also not considered in this study but merits further investigations (27, 52). The impacts of changes in the future anthropogenic Hg emissions are also beyond the scope of this research. Indeed, as inherited by the business-as-usual climate scenario, anthropogenic emissions of Hg emissions are also expected to be high due to fossil fuel consumption (53). Additional Hg emissions may also increase from sources like artisanal and small-scale gold mining (54). A comprehensive picture of the future could be obtained by combining the impacts of anthropogenic emissions and climate-induced changes.

Another source of uncertainty is the methylmercury model used in this study as described by Zhang et al. (9). This model scales the specific methylation rate to OCRR, which is an indicator of microbial activity (8). The empirical methylation rates vary drastically under different environmental conditions (42, 45, 55, 56). There is growing evidence for CH_3_Hg formation in the mixed layer as a result of high productivity (10), which is captured by the model as the modeled OCRR is also high there. In addition, direct measurement of methylation rates in marine water deviates from that predicted by OCRR (55). At a larger scale, the relationship between OCRR and CH_3_Hg concentrations has not been reproduced in ocean basins other than the North Pacific (8, 57), implying the complicated mechanisms of CH_3_Hg formation and deformation compounded by horizontal/vertical transport. This indeed poses challenges for a universal global-scale model and causes significant uncertainties in our predictions of this study (9). Thus, future investigations combining laboratory and in situ incubation experiments should be undertaken to determine the mechanisms of the methylation process mediated by microbes and the dependence of methylation rates and pathways on biogeochemical parameters.

Our projection highlights the potential spur of demethylation potential of CH_3_Hg due to increased light penetration, which is more robust than that for methylation. There is convincing scientific evidence for the photochemical degradation of methylmercury: in situ incubation experiments of both coastal and marine surface waters from the Mediterranean showed that methylmercury could be photochemically degraded (6.4 to 24.5% day^−1^) (10). Experimental studies extended to Pacific waters illustrated the importance of photodemethylation as well and suggested that the photodegradation rate for CH_3_Hg in seawater depends on local incident photon intensity and light attenuation by chlorophyll, DOC, and total suspended materials (11). Lehnherr et al. (42) observed the demethylation of CH_3_Hg in the Arctic Ocean waters and derived a photodemethylation rate constant from isotope analysis data that falls in the same range as previous estimates. Our model also captures the large-scale latitude gradient of MeHg concentrations in the surface seawaters, reflecting the influence of incident solar radiation. Considering the significance of photodemethylation suggested from our results, further studies that examine the association between the photodemethylation rate and shortwave radiation with different wavelengths and the roles of various seawater compositions, especially chlorophyll and dissolved organic matter (or DOC) are needed to better predict the impact of future light environment on the marine Hg cycle.

Our study may suffer from some simplified parameterizations that come from knowledge gaps of some poorly understood processes of Hg biogeochemical cycling, such as biotic and chemical demethylation. Further laboratory work with a focus on mechanisms and their regulation by environmental factors is required to develop a more robust mechanistic parametrization. A better evaluation of the CH_3_Hg bioconcentration through the global ocean also requires more measurements of VCF in environment-relevant concentrations with the consideration of the influence of seawater chemistry (58).

It is important to stress that we cannot convert modeled changes in coastal and open ocean CH_3_Hg levels directly to equivalent changes in the future human CH_3_Hg exposure. The model employed here is not specifically designed to evaluate CH_3_Hg bioconcentrations of higher trophic levels in the marine food chains than plankton (e.g., fish and other aquatic mammals). Thus, the simulated changes in seawater or plankton CH_3_Hg cannot be directly extrapolated to other trophic levels. Indeed, Schartup et al. (4) found a significant increase in the future fish CH_3_Hg with warmer temperatures even holding seawater CH_3_Hg constant due to the changes in fish bioenergetics. They also found divergent trends for different fish species as a result of dietary shifts from the 1970s to the 2000s. Moreover, human exposure to CH_3_Hg through seafood consumption is closely associated with dietary habits and fishing activity, which in turn initiates trophic structure shifts that will drive future seafood CH_3_Hg (59). Other pathways, such as frequent rice ingestion in Hg-contaminated regions (60, 61), contribute to individual exposure to CH_3_Hg as well.

### Implications.

This study sets out to investigate the impact of climate-driven changes on global marine Hg cycling in the hypothesized twenty-first century. Even though the overall prediction is subjected to significant uncertainty, especially regarding the formation of seawater CH_3_Hg due to the limitation of our understanding of its mechanism and influencing factors, we identify several important climate change impact routes (*SI Appendix*, Fig. S8): Seawater Hg^0^ evasion is enhanced by ocean warming but suppressed by decreased wind speed, considerable variation in light environment boosts the photodemethylation of CH_3_Hg, reduced vertical mixing decreases subsurface CH_3_Hg fluxes into the surface ocean, and the loss of sea ice cover facilitates air–sea exchange and also increases photodemethylation. These impacts could also be propagated to the CH_3_Hg concentration in plankton and its potential human exposure.

Climate changes could also exacerbate marine Hg contamination. Previous studies also indicated the possibility of Hg release as a consequence of glaciers melting and permafrost thawing (62), and the released Hg poses a potential threat to polar waters and the global environment (37, 63, 64). These results reinforce the need to better characterize interactions of the marine Hg cycle with processes across multiple scales. A natural progression of this work is to further investigate the feedback within the biogeochemistry cycle of Hg via coupled earth system models.

Taken together, there is still a great technical challenge to predict the future marine Hg cycles, and the uncertainty arises from our mechanical understanding of the Hg cycle itself, the direction and magnitude of climate change and incurred environmental changes, as well as the future path of anthropogenic emissions. Our results are thus diagnostic, yet offer insight into synergisms/antagonisms on marine Hg cycling among ocean warming, light environment, circulation change, ocean acidification, and other ecological and biogeochemical stressors. This may present a perceptible way of identifying future human exposure to CH_3_Hg and improving relevant regulatory strategies in the context of global climate change.

## Methods

### MITgcm-Hg.

We apply a global three-dimensional ocean model for mercury (Hg) to calculate the impacts of climate change on the marine Hg cycle. We use the MITgcm-Hg model, which comprehensively accounts for the physical, chemical, and biological processes that control marine Hg biogeochemistry. MITgcm-Hg was described and tested against measurement data by Zhang et al. (9). The model simulates the transport, transfer, and transformation of Hg in the global ocean in inorganic and organic forms, including the redox transformation between elemental Hg (Hg^0^) and inorganic divalent Hg (Hg^II^), methylation and demethylation of monomethylmercury (CH_3_Hg) and dimethylmercury [(CH_3_)_2_Hg], air–sea exchange of Hg^0^ and (CH_3_)_2_Hg, partitioning between dissolved and particulate phases, particle-bound Hg sinking, and CH_3_Hg bioaccumulation within marine plankton food webs. Further details can be found in *SI Appendix, Methods* and Table S5.

We apply the MITgcm-Hg model at a horizontal resolution of 2° × 2.5° with 22 vertical levels down to 5,192.5 m, consistent with the Integrated Global System Model (IGSM) framework (65) that provides the physical state of the global ocean for the model. The ocean biogeochemistry and ecosystem variables for the Hg model are from Dutkiewicz et al. (23) and are simulated by the Darwin marine ecosystem model, which has the same resolution as IGSM and is driven by the archived ocean physics field from it. Other physical variables at the air–sea interface are obtained from either reanalysis data for “present-day” conditions (9) or Coupled Model Intercomparison Project Phase 5 (https://esgf-node.llnl.gov/search/cmip5) multi-model mean for the future scenario. Details of the modeling system are described in *SI Appendix*, *Methods* and Fig. S5 shows the model coupling configuration for this study. The model is run for 10 y for each scenario simulation (1991 to 2000 for the present-day or 2091 to 2100 for the future). This setup allows Hg species to achieve a steady state for responses to changes in ocean physics and biogeochemistry (9). We use the result of the last year (i.e., the years 2000 and 2100) for data analysis. The model's initial condition is regridded from the result obtained by previous ocean modeling work (66). The upper boundary conditions of the MITgcm-Hg (i.e., atmospheric Hg concentrations and deposition fluxes) are from the GEOS-Chem atmospheric Hg simulation (67) and are held to repeat a seasonal cycle. Anthropogenic Hg emissions are kept unchanged to diagnose the effect of climate change.

### Experiment Design.

To unravel the effects of individual factors on the marine Hg cycle, a suite of simulations is performed in the hypothesized twenty-first century under the business-as-usual scenario. First, we simulate the marine Hg cycling from 1991 to 2000 using the ocean physical state results and Darwin model results from the same time period and other physical variables (surface downward shortwave radiation, near-surface wind speed, and sea ice coverage) that are representative of the present-day conditions to serve as the present-day baseline condition. Each experiment in the future climate change scenario then simulates the year 2091 to 2100. Separating the effects of different factors is achieved by alternatively changing merely one factor to the future condition with other variables repeating present-day conditions. These factors specifically include ocean velocity and mixing, seawater temperature, surface downward shortwave radiation, near-surface wind speed, sea ice coverage, marine plankton community structure, chlorophyll concentration, DOC concentration, POC concentration, and OCRR. A total of 10 sensitivity experiments are performed and denoted as *circulation, temperature, radiation, wind speed, sea ice, community structure, Chl, DOC, POC, and OCRR*, in which the above variables are altered, respectively. The experiment *community structure* allows variables of phytoplankton biomass, zooplankton grazing fluxes for each phytoplankton category, and mortality fluxes of zooplankton, which reflect the structure of planktonic food web, to change to future conditions. Experiment *all* represents that all these factors above are altered to the future conditions.

To further distinguish the effects of ocean acidification and physical factors (e.g., circulation, temperature) on ocean biogeochemistry (5), we supplement experiments denoted as *community_physics, Chl_physics, DOC_physics, POC_physics,* and *OCRR_physics*, in which “*_physics*” indicates that *p*CO_2_ was held at present-day conditions while all other climate fields are allowed to change. Similarly, the scenario with a subscript “*_acid*” is ascribed to ocean acidification (*SI Appendix*, Table S2).

## Data, Materials, and Software Availability

Modeling results and analysis scripts in this study are deposited in Mendeley Data: https://doi.org/10.17632/br2psms8b9.1. Other resources of public accessible data are given in the article and/or *SI Appendix*.

## Supplementary Material

Appendix 01 (PDF)
