# Supplementary material for: Climate-driven changes of global marine mercury cycles in 2100
Source: Proc Natl Acad Sci U S A. 2023 Jan 3;120(2):e2202488120. doi: 10.1073/pnas.2202488120 (PMC9926249; doi:10.1073/pnas.2202488120)
Supplement: Supplementary file 1 — Appendix 01 (PDF) [file pnas.2202488120.sapp.pdf]

Supporting Information for

Climate-driven changes of global marine mercury cycles in 2100

Yujuan Wang, Peipei Wu, and Yanxu Zhang*

School of Atmospheric Sciences, Nanjing University, Nanjing, Jiangsu 210023, China

*Correspondence to Y.Z. (zhangyx@nju.edu.cn)

Methods

MITgcm-Hg MITgcm-Hg model simulates the exchange of volatile Hg species [Hg^0 and $(CH_3)_2Hg$] at the air-sea interface based on the concentration gradient across the interface, uncovered sea surface area, and piston velocity calculated as a function of wind speed. Partitioning of Hg^{II} and CH_3Hg between dissolved and particulate phase is modeled based on partition coefficients and POC concentration (including detritus and living plankton). The sinking flux of particulate Hg species are then calculated according to the export flux curves for POC in the water column.

The inorganic Hg chemistry in MITgcm-Hg includes photochemical and biological redox conversions between dissolved Hg^0 and Hg^{II} in the oceanic mixed layer and biologically mediated redox reactions in the subsurface ocean. The dark oxidation of dissolved Hg^0 in the mixed layer is also included (1). The formation rates of methylated Hg are parameterized according to microbial remineralization of organic carbon. The photochemical and dark demethylation processes are included. The rate constants of photochemical reactions are scaled by the shortwave radiation intensity attenuated by ocean pigments (chlorophyll and dissolved organic carbon). The dark demethylation rate is calculated as a function of seawater temperature. CH_3Hg uptake by phytoplankton is simulated with volume concentration factors as a function of cell size and seawater DOC concentration (2, 3). The trophic transfer of CH_3Hg to zooplankton is modeled with the processes including zooplankton grazing, excretion, and mortality. The detailed parameterizations used in the model are listed in Table S5.

Ocean physical data The future ocean physical variables (including velocities, mixing, temperature, and salinity) in both present-day and future experiments are taken from Dutkiewicz et al. (4) and Sokolov et al. (5), which are based on the MIT Integrated Global System Model (IGSM) framework (Fig. S5). This model system is of intermediate complexity, including a three-dimensional ocean model and a two-dimensional atmosphere model. It also has a terrestrial component that dynamically updates greenhouse gas emissions. The model is spun up for 2000 years before simulating the period 1860-2100. The atmospheric greenhouse gas concentrations are specified with observed values during historical (1860-1990) time, but from 1991 to 2100, anthropogenic emissions for a 'business as usual' scenario [close to IPCC AR5 Representative Concentration Pathways (RCP) 8.5 scenario] is used.

Marine ecosystem model The ocean biogeochemistry and ecosystem variables are from Dutkiewicz et al. (6), which is simulated by the Darwin marine ecosystem model. The model has the same resolution as IGSM and is driven by the archived ocean physics field from it (Fig. S5). This three-dimensional ecology model simulates the cycling of nutrients, including carbon, nitrogen, phosphorus, iron, and silica. It also simulates a marine ecosystem including six categories of phytoplankton functional groups (diatoms, other large, diazotrophs, coccolithophores, *Prochlorococcus,* and *Synechococcus*; 16 species for each group and 96 in sum) and two herbivorous zooplankton grazers with different sizes (small and large). Here we use the monthly mean results of organic carbon concentration, chlorophyll concentration, phytoplankton biomass,

carbon remineralization rates, zooplankton grazing fluxes for each phytoplankton category, and mortality fluxes of zooplankton to drive the global ocean model for Hg.

CMIP5 products Other physical variables (surface downward shortwave radiation, near-surface wind speed, and sea ice coverage) are from either reanalysis data for “present-day” conditions (7) or CMIP5 (Coupled Model Intercomparison Project Phase 5, https://esgf-node.llnl.gov/search/cmip5) multi-model mean for the future scenario. We use the climate projections for a total of 22 CMIP5 models (Table S1). We make a selection of ensemble member “r1i1p1” from the RCP 8.5 experiment of each CMIP5 model. Model output on each model native grid is regridded to fit the Hg model using bilinear interpolation. We use monthly mean data of the year 2100, repeated for ten years in the future scenario simulations. To better represent future climate scenarios, all models are given equal weight in the multi-model mean.

Global riverine nutrient load We use the riverine nutrient discharge results generated from Global Nutrient Export from Watersheds 2 (NEWS 2) model (8, 9) to derive eutrophication-induced changes in the marine ecosystem. Export of dissolved inorganic nitrogen and phosphorus (DIN, DIP), dissolved organic carbon, nitrogen, and phosphorus (DOC, DON, DOP), particulate organic carbon (POC), particulate nitrogen and phosphorus (PN and PP), and dissolved silica (DSi) at river mouths to the ocean coast is used as input in the Darwin marine ecosystem model. Data from the year 2000 are used for the present-day scenario and data from the year 2050 for the future scenario.

Table S1: The CMIP5 Earth system models used in this study.

**Modeling Center**	**Model**	**Resolution**
Commonwealth Scientific and Industrial Research Organization (CSIRO)	ACCESS 1.0	192×72
	ACCESS 1.3	192×72
Canadian Centre for Climate Modelling and Analysis	CanESM 2	128×64
Centro Euro-Mediterraneo per I Cambiamenti Climatici	CMCC-CESM	480×240
	CMCC-CM	480×240
	CMCC-CMS	192×48
Météo-France/Centre National de Recherches Météorologiques	CNRM-CM5	256×128
CSIRO	CSIRO-Mk3-6-0	192×96
Institute of Atmospheric Physics, Chinese Academy of Sciences (LASG/IAP)	FGOALS s2	128×64
U.S. Department of Commerce/National Oceanic and Atmospheric Administration (NOAA)/Geophysical Fluid Dynamics Laboratory (GFDL)	GFDL-CM3	144×90
	GFDL-ESM2G	144×90
	GFDL-ESM2M	144×90
National Aeronautics and Space Administration (NASA)/Goddard Institute for Space Studies (GISS)	GISS-E2-H	144×90
	GISS-E2-H-CC	144×90
	GISS-E2-R	144×90
	GISS-E2-R-CC	144×90
Met Office Hadley Center	HadGEM2-AO	192×72
	HadGEM2-CC	192×72
	HadGEM2-ES	192×72
Institut Pierre-Simon Laplace	IPSL-CM4A-MR	144×70
	IPSL-CM5A-LR	96×96
	IPSL-CM5B-LR	96×96

Table S2: Experiment lists.

	Annotation/Name	Supplementary experiments	
		Physical factors related changes	Ocean acidification related changes
**Present days**	base, 2000, or present-day	\	\
**Physical changes**	circulation	\	\
	temperature	\	\
	radiation	\	\
	wind speed	\	\
	seaice	\	\
**Biological changes**	community structure	community_physics	community_acid
**Biogeochemical changes**	Chl	Chl_physics	Chl_acid
	DOC	DOC_physics	DOC_acid
	POC	POC_physics	POC_acid
	OCRR	OCRR_physics	OCRR_acid
**All factors**	all	\	\

Table S3: The average concentrations of inorganic Hg, seawater CH_3Hg, and CH_3Hg in phytoplankton in the surface ocean (0-100 m) and in the subsurface ocean (100-1000 m) in all the simulations (unit: fM). The average of the coastal grids is given in brackets.

	**Annotation/Name**	**Surface ocean**			**Subsurface ocean**	
		Inorganic Hg	Seawater CH_3Hg	Phytop. CH_3Hg	Inorganic Hg	Seawater CH_3Hg
**Present-day**	2000	507.2 (490.7)	44.7 (55.9)	1.7 (2.0)	984.4 (1084.0)	188.8 (244.7)
**Physical changes**	circulation	499.1 (484.0)	43.4 (52.8)	1.6 (1.9)	984.8 (1085.0)	193.6 (246.0)
	temperature	473.4 (462.4)	42.1 (53.0)	1.6 (1.9)	984.5 (1088.8)	179.8 (233.8)
	radiation	509.4 (495.8)	40..8 (48.9)	1.5 (1.7)	985.9 (1087.8)	187.6 (242.9)
	wind speed	539.3 (525.7)	47.1 (59.6)	1.8 (2.1)	1001.0 (1097.4)	191.6 (247.1)
	seaice	507.6 (489.6)	39.8 (45.9)	1.6 (1.7)	985.2 (1085.3)	188.3 (243.7)
**Biological changes**	comm. struc.	499.0 (481.7)	43.7 (54.3)	1.8 (2.2)	980.5 (1079.6)	187.7 (243.2)
	community_physics	498.4 (481.5)	43.7 (54.4)	1.7 (2.0)	980.5 (1079.7)	187.7 (243.2)
**Biogeoche-mical changes**	Chl	513.8 (493.6)	30.1 (38.6)	1.2 (1.3)	998.0 (1102.7)	184.9 (238.5)
	Chl_ physics	513.8 (493.6)	30.2 (38.6)	1.2 (1.3)	998.0 (1102.5)	184.9 (238.6)
	DOC	506.6 (489.4)	45.7 (57.6)	1.7 (2.0)	983.5 (1082.0)	189.2 (245.8)
	DOC_ physics	506.8 (489.6)	45.5 (57.6)	1.7 (2.0)	983.7 (1082.3)	189.2 (245.7)
	POC	513.2 (462.8)	44.0 (53.3)	1.8 (2.0)	943.8 (1021.3)	179.9 (232.5)
	POC_ physics	513.0 (462.2)	44.0 (53.2)	1.8 (2.0)	943.4 (1020.5)	179.8 (232.3)
	OCRR	505.3 (475.9)	40.1 (56.0)	1.5 (1.8)	1035.9 (1144.8)	156.7 (201.4)
	OCRR_ physics	507.0 (477.8)	37.8 (53.8)	1.4 (1.8)	1037.7 (1145.8)	155.6 (201.0)
**All factors**	all	501.6 (455.4)	20.6 (23.8)	1.0 (1.2)	1008.6 (1111.7)	143.2 (176.9)

Table S4: The average concentrations of inorganic Hg, seawater CH_3Hg, and CH_3Hg in plankton in the surface ocean (0-100 m) and in the subsurface ocean (100-1000 m) in the experiments of riverine nutrient discharge changes (unit: fM). The averages of coastal grids are given in brackets.

**River nutrient load scenario**	**Surface ocean**			**Subsurface ocean**	
	Inorganic Hg	Seawater CH_3Hg	Plankton CH_3Hg	Inorganic Hg	Seawater CH_3Hg
2000	470.7 (412.6)	47.6 (98.5)	4.1 (8.9)	980.0 (1032.5)	190.6 (291.7)
2100	470.7 (412.5)	47.6 (98.5)	4.1 (9.0)	980.1 (1033.1)	190.5 (291.3)

Table S5: Model description of the main processes in the ocean Hg model. Source: Zhang et al. (1, 7).

Description	Equation or value
Photooxidation rate from Hg^0 to Hg^{II}, s^{-1}	$k_{photo-ox} = 6.6\times10^{-6}\times hv$
Photoreduction rate from Hg^{II} to Hg^0, s^{-1}	$k_{photo-red} = 1.6\times10^{-6}\times hv$
Biological oxidation rate from Hg^0 to Hg^{II}, s^{-1}	$k_{bio-ox} = 140\times \mathrm{OCRR}$
Biological reduction rate from Hg^{II} to Hg^0, s^{-1}	$k_{bio-red} = 86\times \mathrm{OCRR}$
Dark oxidation rate from Hg^0 to Hg^{II}, s^{-1}	$k_{dark-ox} = 1.0\times10^{-7}$
Methylation rate from Hg^{II} to CH_3Hg, s^{-1}	$k_{m1} = 4.4\times10^{-7}\times \mathrm{OCRR}$
Methylation rate from CH_3Hg to $(CH_3)_2Hg$, s^{-1}	$k_{m2} = 9.3\times10^{-9}$
Photodemethylation rate from CH_3Hg to Hg^{II}, s^{-1}	$k_{dm1} = 8.0\times10^{-8}\times hv$
Demethylation rate from $(CH_3)_2Hg$ to CH_3Hg, s^{-1}	$k_{dm2} = 1.9\times10^{-8}+3.8\times10^{-9}\times hv$
Dark demethylation rate from CH_3Hg to Hg^0, s^{-1}	$k_{dm3} = 1.1\times10^{-8}\times\exp\left(-5500\times\left(\frac{1}{T}-\frac{1}{293.15}\right)\right)$
Volume concentration factor of CH_3Hg between phytoplankton and seawater	$\mathrm{VCF} = 2.8\times10^{6}\times\frac{1}{d}\times e^{-0.008\cdot DOC}$
The transfer rate of CH_3Hg from *i*th phytoplankton to *j*th zooplankton via dietary uptake, mol m^{-3} s^{-1}	$F_{graze}^{CH_3Hg}\big\|_{i,j} = A_{ED}\times F_{graze}^{Carbon}\big\|_{i,j}\times\frac{\mathrm{CH_3Hg}_{\mathrm{phy}}^{i}}{Phy_i}$
The flux of CH_3Hg that is released back to seawater from the *j*th zooplankton due to mortality, mol m^{-3} s^{-1}	$F_{mort}^{CH_3Hg}\big\|_{j} = F_{mort}^{Carbon}\big\|_{j}\times\frac{\mathrm{CH_3Hg}_{zoo}^{j}}{Zoo_j}$
Elimination rate of CH_3Hg from zooplankton, s^{-1}	$k_{elim} = 3.8\times10^{-8}\times M_{zoo}^{-0.195}\times e^{0.0066T}$
Incoming shortwave radiation flux, W m^{-2}	hv
Organic carbon remineralization rate, mmol m^{-3} day^{-1}	OCRR
Seawater temperature, K	T
Plankton cell diameter, μm	d
Dissolved organic carbon concentration in seawater, μM	DOC
Assimilation efficiency of zooplankton for CH_3Hg	$A_{ED} = 0.6$
Biomass (in carbon content) of the *i*th phytoplankton grazed by the *j*th zooplankton, mol m^{-3} s^{-1}	$F_{graze}^{Carbon}\big\|_{i,j}$
Rate of carbon release to seawater following the *j*th zooplankton mortality, mol m^{-3} s^{-1}	$F_{mort}^{Carbon}\big\|_{j}$
CH_3Hg concentrations in phytoplankton and zooplankton, mol m^{-3}	$\mathrm{CH_3Hg}_{\mathrm{phy}}^{i}$, $\mathrm{CH_3Hg}_{zoo}^{j}$
Biomass (in carbon content) concentration of phytoplankton and zooplankton, mol m^{-3}	Phy_i, Zoo_j
Wet weight of zooplankton cell, g	M_{zoo}

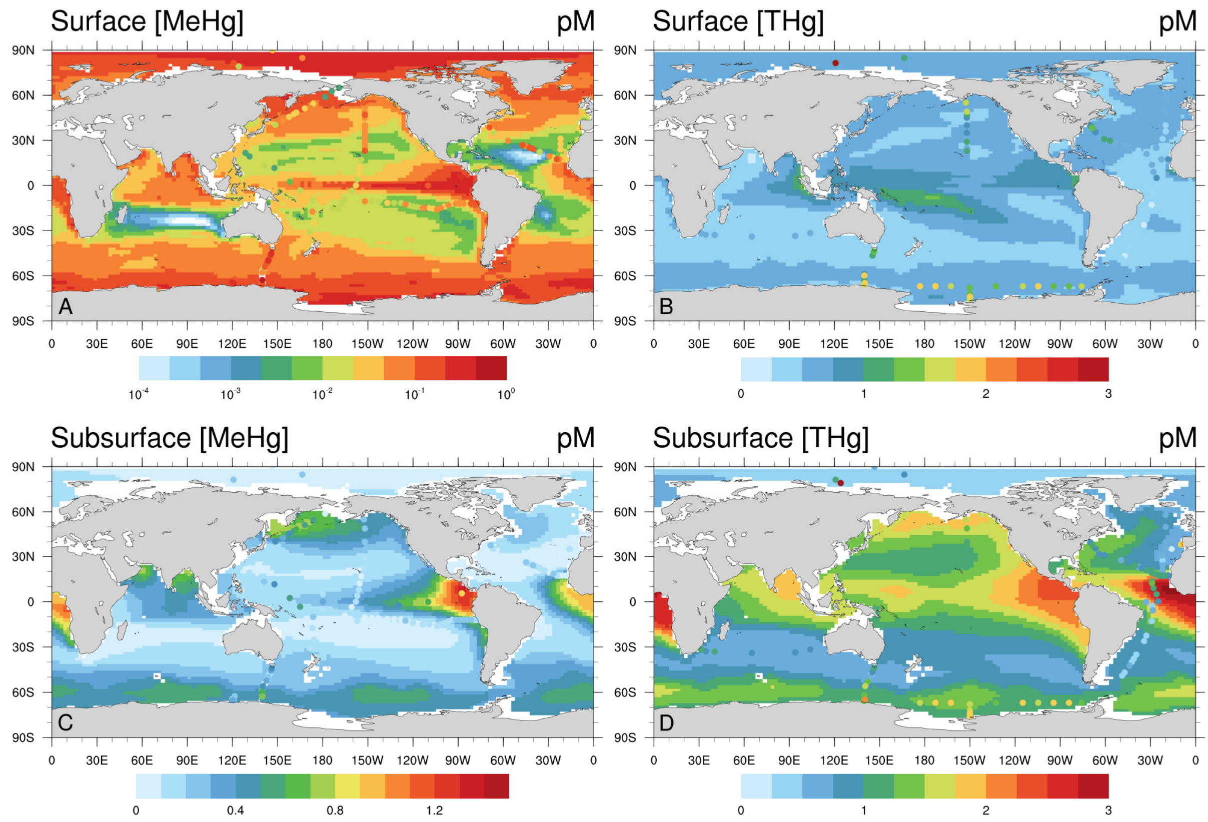

Figure S1: Comparison of model results (contour fill) and observations (filled circles). Total methylated Hg [MeHg, i.e., CH_3Hg and $(CH_3)_2Hg$] concentration in the *A* surface and *C* subsurface ocean. Total Hg (THg) concentration in the *B* surface and *D* subsurface ocean. Observation data used here is summarized in Zhang, et al. (2020).

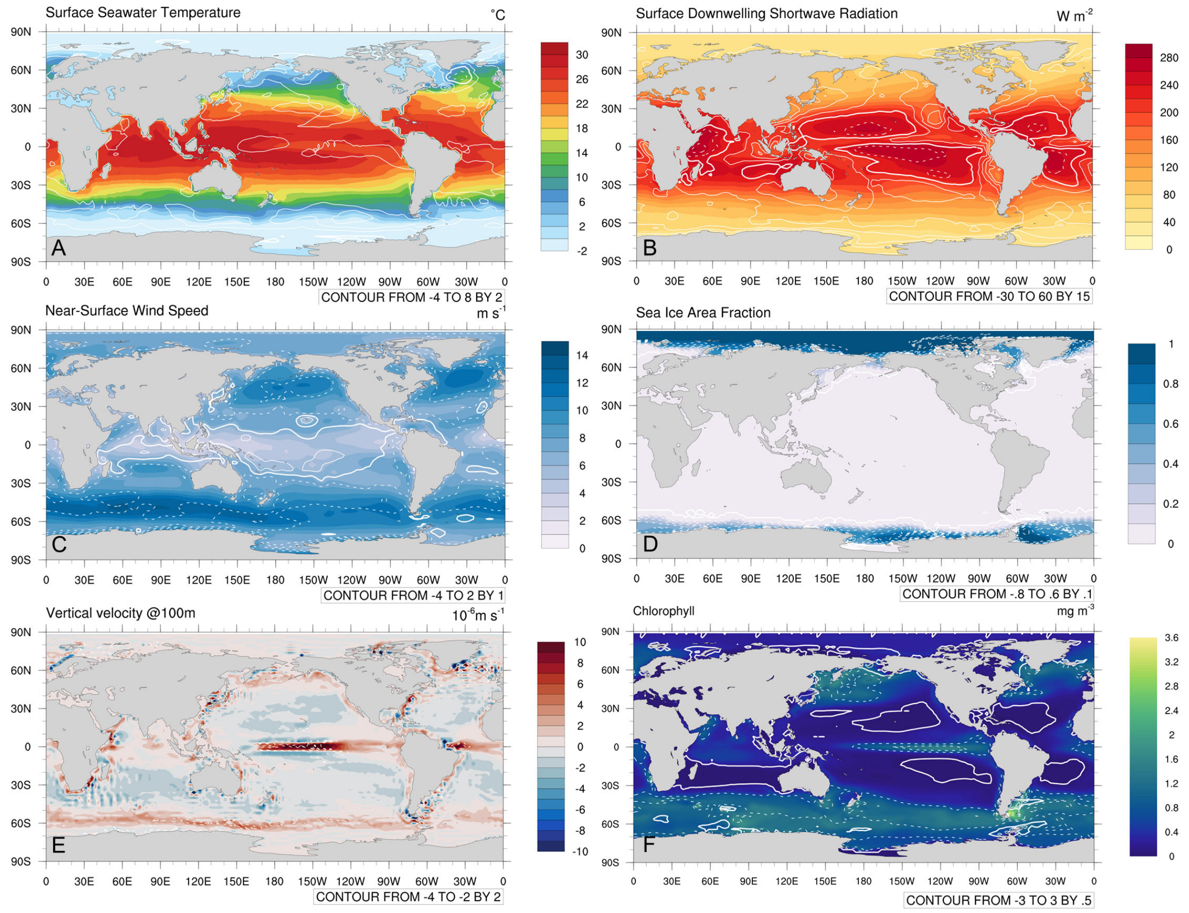

Figure S2: Physical condition and chlorophyll concentration changes between the future scenario and present-day. *A* Annual mean (shading) of present-day surface seawater temperature and differences (contours) between 2100 and present days. Solid contours indicate increase (positive values) and dashed contours indicate decrease (negative values). Note that the thick solid line is the zero contour. Same plot for *B* surface downward shortwave radiation, *C* near-surface wind speed, *D* sea ice area fraction, *E* vertical velocity at 100 m, and *F* chlorophyll concentration in upper 100 m.

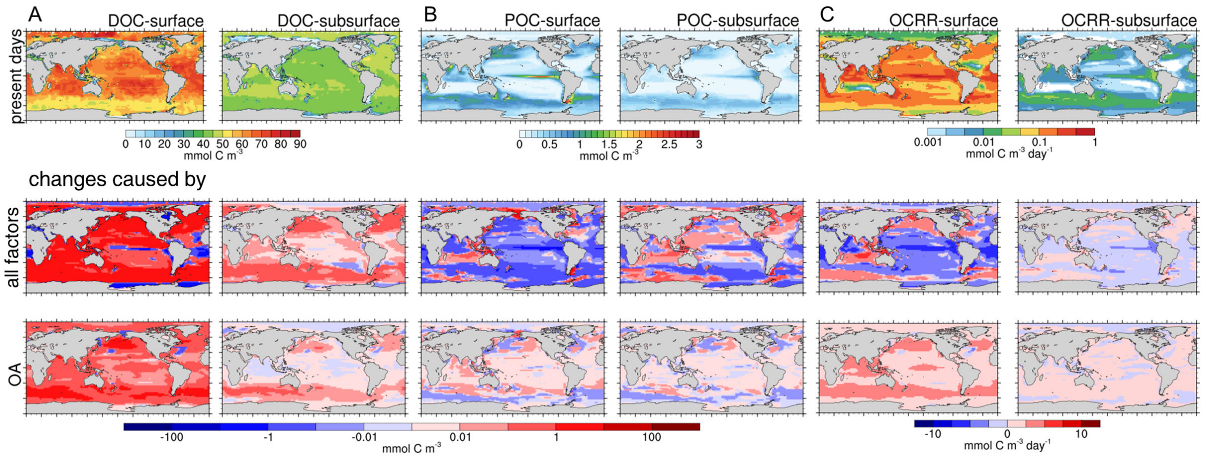

Figure S3: Biogeochemical changes between the future scenario and present-day. Present-day dissolved organic carbon (DOC) concentration (1st row) in the surface (upper 100m, left column) and subsurface (100-1000m, right column) ocean, differences caused by all factors between 2100 and present-day (2nd row), and the changes caused by ocean acidification only (3rd row) (group *A*, i.e. the first two columns), Same plot for particulate organic carbon (POC) concentration (group *B*, i.e. the middle two columns); particulate organic carbon remineralization rate (OCRR) (group *C*, i.e. the right two columns).

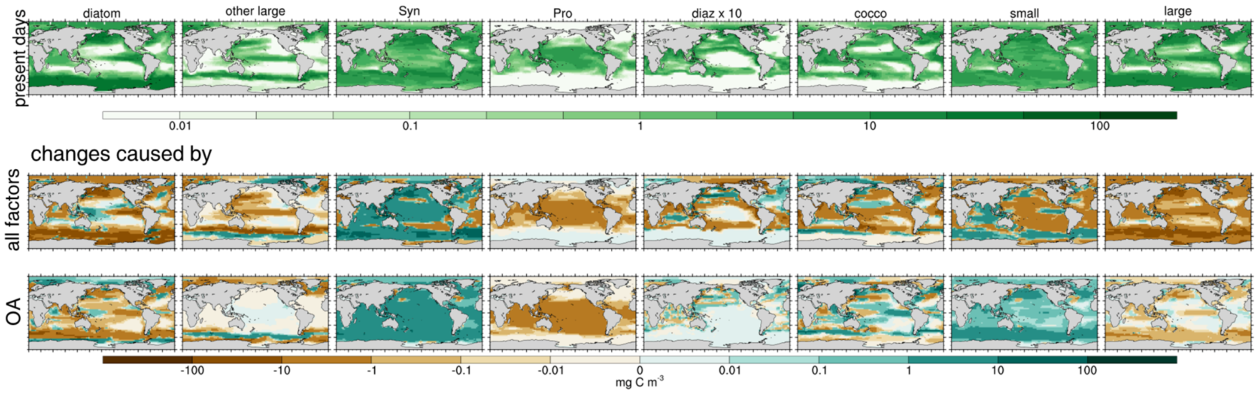

Figure S4: Biological changes between the future scenario and present-day. Present-day biomass of six individual phytoplankton functional groups (diatom, other large, *Synechococcus*, *Prochlorococcus*, diazotrophs, coccolithophores) and two herbivorous zooplankton types (small zooplankton and large zooplankton) (1st row) in the surface ocean (upper 100m), differences caused by all factors between 2100 and present-day (2nd row), and the effect of ocean acidification only (3rd row).

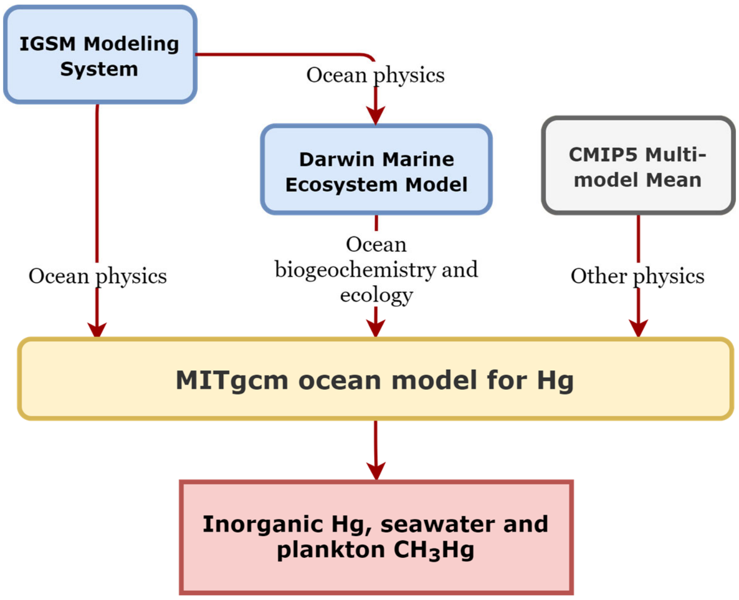

Figure S5: Schematic of model coupling method in this study.

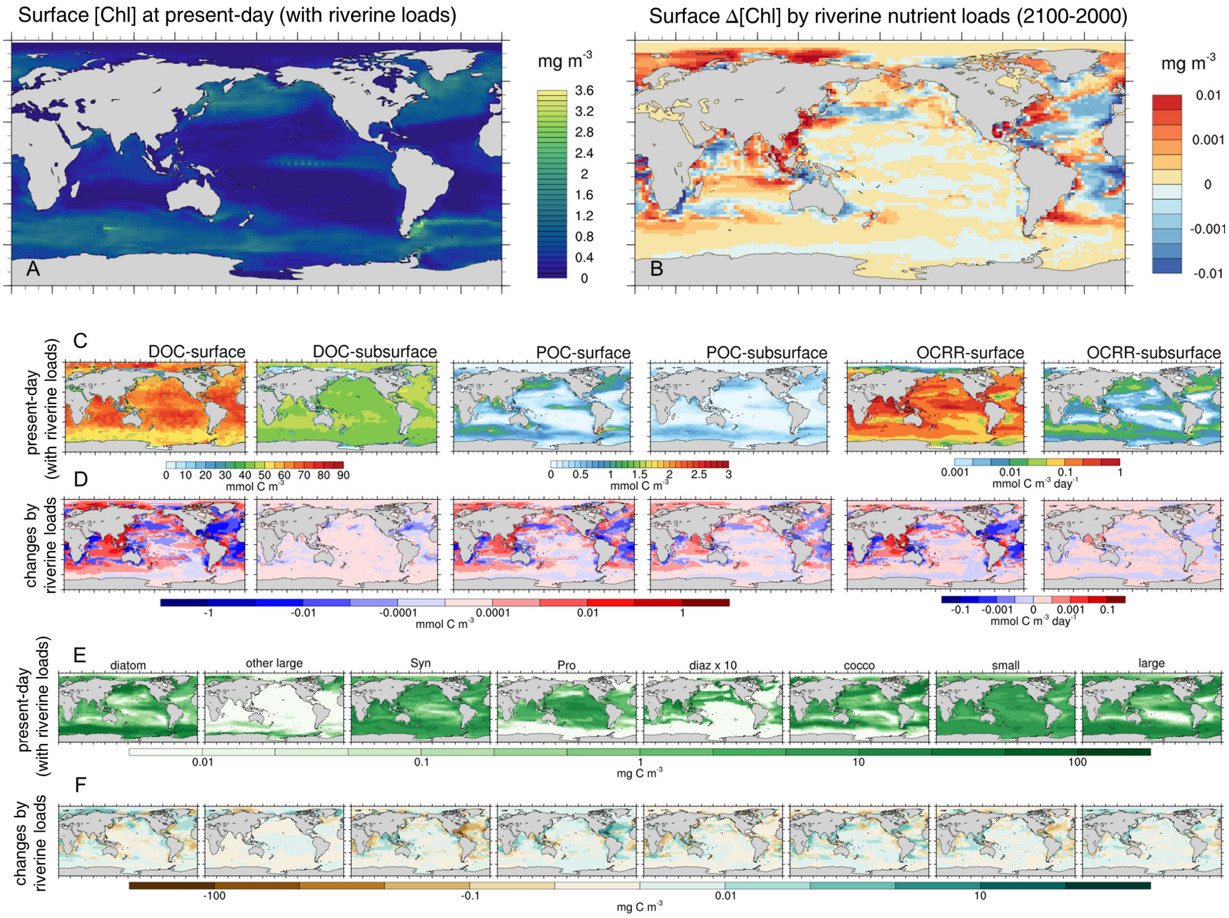

Figure S6: Biogeochemical changes caused by the different riverine nutrient discharges between 2100 and present-day. *A* Present-day chlorophyll concentration in the surface (upper 100m) ocean, and *B* differences between 2100 and present-day. (row *C*) Present-day dissolved organic carbon (DOC) concentration in the surface (upper 100m, left column) and subsurface (100-1000m, right column) ocean, differences between 2100 and present-day (row *D*). Same plot for particulate organic carbon (POC) concentration and particulate organic carbon remineralization rate (OCRR)**.** Present-day biomass of six individual phytoplankton functional groups and two herbivorous zooplankton types (*E* row) in the surface ocean, differences between 2100 and present-day (*F* row).

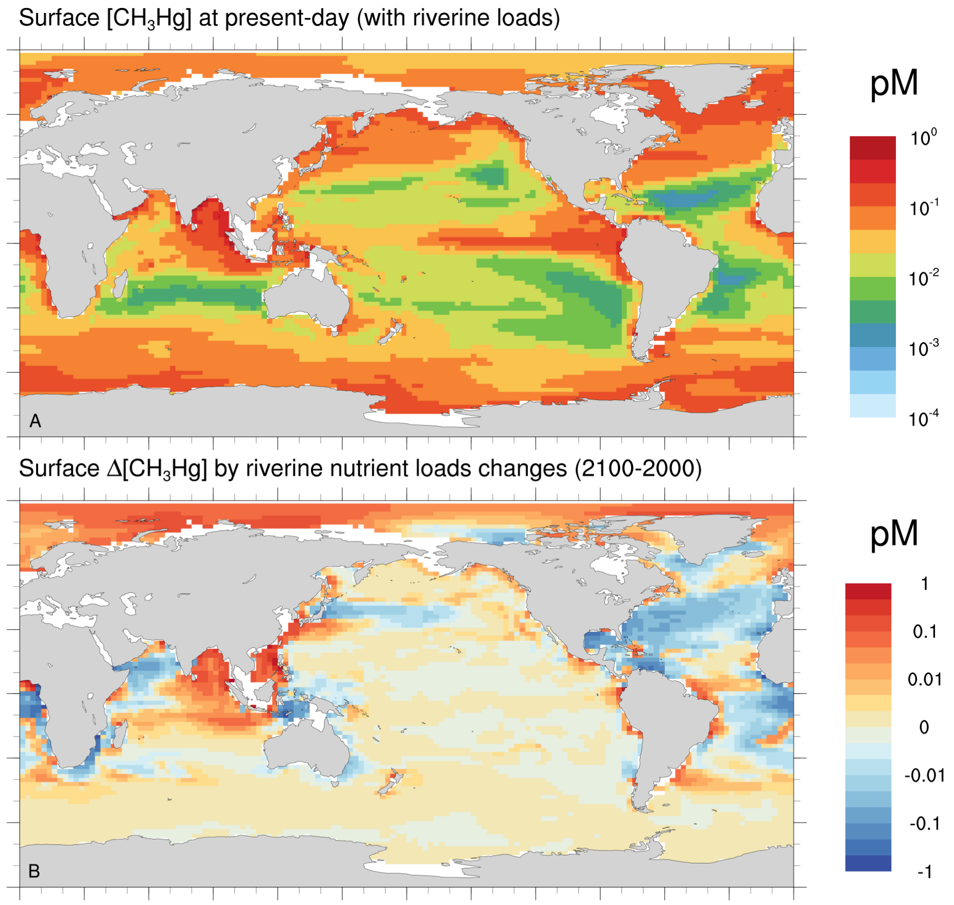

Figure S7: Modeled seawater CH_3Hg concentration caused by the different riverine nutrient discharges between 2100 and present-day. Model results of seawater CH_3Hg concentration with the eutrophication effects in present days (*A*) and differences between 2100 and present-day (*B*).

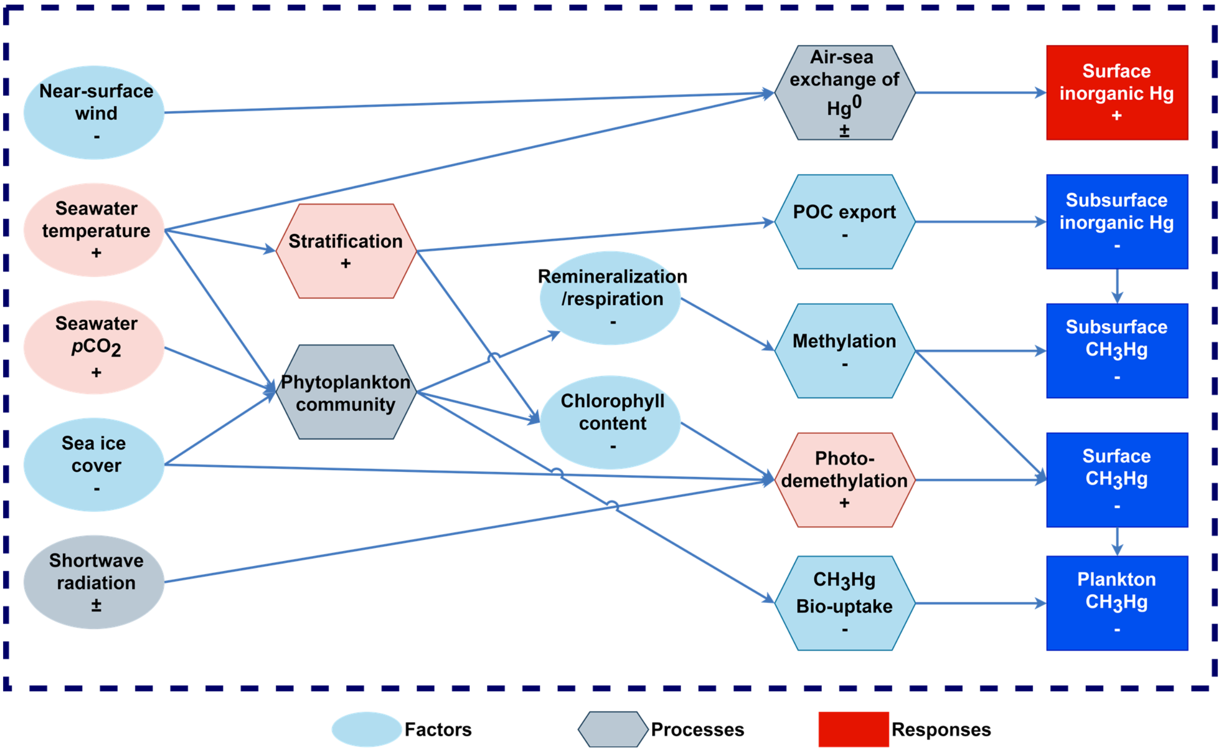

Figure S8: Schematic representation of the effects of climate change impacts on marine mercury cycling.

SI References

1. Y. Zhang, L. Jaeglé, L. Thompson, Natural biogeochemical cycle of mercury in a global three-dimensional ocean tracer model: Global 3D ocean model for mercury. *Glob. Biogeochem. Cycles* **28**, 553–570 (2014).
2. C.-S. Lee, N. S. Fisher, Methylmercury uptake by diverse marine phytoplankton. *Limnol. Oceanogr.* **61**, 1626–1639 (2016).
3. A. T. Schartup, *et al.*, A Model for Methylmercury Uptake and Trophic Transfer by Marine Plankton. *Environ. Sci. Technol.* **52**, 654–662 (2018).
4. S. Dutkiewicz, J. R. Scott, M. J. Follows, Winners and losers: Ecological and biogeochemical changes in a warming ocean: WINNERS AND LOSERS. *Glob. Biogeochem. Cycles* **27**, 463–477 (2013).
5. A. P. Sokolov, *et al.*, Probabilistic Forecast for Twenty-First-Century Climate Based on Uncertainties in Emissions (Without Policy) and Climate Parameters. *J. Clim.* **22**, 5175–5204 (2009).
6. S. Dutkiewicz, *et al.*, Impact of ocean acidification on the structure of future phytoplankton communities. *Nat. Clim. Change* **5**, 1002–1006 (2015).
7. Y. Zhang, A. L. Soerensen, A. T. Schartup, E. M. Sunderland, A Global Model for Methylmercury Formation and Uptake at the Base of Marine Food Webs. *Glob. Biogeochem. Cycles* **34**, e2019GB006348 (2020).
8. E. Mayorga, *et al.*, Global Nutrient Export from WaterSheds 2 (NEWS 2): Model development and implementation. *Environ. Model.*, 17 (2010).
9. S. P. Seitzinger, *et al.*, Global river nutrient export: A scenario analysis of past and future trends. *Glob. Biogeochem. Cycles*, 16 (2010).
